# Arbuscular mycorrhiza induces low oxidative burst in drought-stressed walnut through activating antioxidant defense systems and heat shock transcription factor expression

**DOI:** 10.3389/fpls.2022.1089420

**Published:** 2022-11-29

**Authors:** Wen-Ya Ma, Qiu-Yun Qin, Ying-Ning Zou, Kamil Kuča, Bhoopander Giri, Qiang-Sheng Wu, Abeer Hashem, Al-Bandari Fahad Al-Arjani, Khalid F. Almutairi, Elsayed Fathi Abd_Allah, Yong-Jie Xu

**Affiliations:** ^1^ Tibet Plateau Walnut Industry Research Institute/College of Horticulture and Gardening, Yangtze University, Jingzhou, Hubei, China; ^2^ Department of Chemistry, Faculty of Science, University of Hradec Kralove, Hradec Kralove, Czechia; ^3^ Department of Botany, Swami Shraddhanand College, University of Delhi, New Delhi, India; ^4^ Botany and Microbiology Department, College of Science, King Saud University, Riyadh, Saudi Arabia; ^5^ Plant Production Department, College of Food and Agricultural Sciences, King Saud University, Riyadh, Saudi Arabia; ^6^ Hubei Key Laboratory of Economic Forest Germplasm Improvement and Resources Comprehensive Utilization, Hubei Collaborative Innovation Center for the Characteristic Resources Exploitation of Dabie Mountains, Huanggang Normal University, Huanggang, China; ^7^ Hubei Academy of Forestry, Wuhan, China

**Keywords:** arbuscular mycorrhiza, heat shock transcription factor, reactive oxygen species, symbiosis, water stress

## Abstract

Arbuscular mycorrhizal fungi (AMF) have important roles in enhancing drought tolerance of host plants, but it is not clear whether and how AMF increase drought tolerance in walnut (*Juglans regia*). We hypothesized that AMF could activate antioxidant defense systems and heat shock transcription factors (*Hsfs*) transcription levels to alleviate oxidative damage caused by drought. The walnut variety ‘Liaohe No. 1’ was inoculated with *Diversispora spurca* and exposed to well-watered (WW, 75% of the maximum soil water capacity) and drought stress (DS, 50% of the maximum soil water capacity) for 6 weeks. Plant growth, antioxidant defense systems, and expressions of five *JrHsfs* in leaves were studied. Such drought treatment inhibited root mycorrhizal colonization, while plant growth performance was still improved by AMF inoculation. Mycorrhizal fungal inoculation triggered the increase in soluble protein, glutathione (GSH), ascorbic acid (ASC), and total ASC contents and ascorbic peroxidase and glutathione reductase activities, along with lower hydrogen peroxide (H_2_O_2_), superoxide anion radical (O_2_^•−^), and malondialdehyde (MDA) levels, compared with non-inoculation under drought. Mycorrhizal plants also recorded higher peroxidase, catalase, and superoxide dismutase activities than non-mycorrhizal plants under drought. The expression of *JrHsf03*, *JrHsf05*, *JrHsf20*, *JrHsf22*, and *JrHsf24* was up-regulated under WW by AMF, while the expression of *JrHsf03*, *JrHsf22*, and *JrHsf24* were up-regulated only under drought by AMF. It is concluded that *D*. *spurca* induced low oxidative burst in drought-stressed walnut through activating antioxidant defense systems and part *Hsfs* expressions.

## Introduction

Walnuts (*Juglans regia* L.) are an important nut crop in the world, with the second highest yield of nut crops (Behrooz et al., 2019). Walnut kernels can not only be consumed directly, but also include a large amount of unsaturated fatty acids and a variety of active ingredients (Luca et al., 2018). Among them, the high content of polyphenols in walnuts makes them effective as antioxidants and free radical scavengers (Ebrahimzadeh et al., 2013). Walnut trees are influenced by soil drought stress (DS) because of their high water demand (Vahdati et al., 2009).

Arbuscular mycorrhizal fungi (AMF) establish symbiotic associations with various plants (Wu et al., 2013). AMF can help the host to acquire nutrients from the soil, especially difficult-to-move elements, and thus increase plant growth (Ho-Plágaro et al., 2021). Studies indicated that AMF inoculation enhanced drought tolerance of host plants through various underlying mechanisms, as outlined by Cheng et al. (2021). One important mechanism is the ability of AMF to mitigate oxidative burst by enhancing antioxidant defense systems of the host (Zou et al., 2021). The population of AMF has been observed in rhizosphere of walnuts (Ma et al., 2021), and AMF inoculation contributed to walnut growth (Wang, 2015; Huang et al., 2020). Combining AMF (*Glomus fasciculatus*) inoculation with foliar fertilization would increase plant growth as well as the survival of walnuts (Ponder, 1984). In addition, AMF (*Diversispora spurca*) inoculation accelerated nutrient uptake of walnuts such as P and K (Huang et al., 2020; Thioye et al., 2022). Potted studies had shown the role of AMF in drought tolerance of walnut plants. Behrooz et al. (2019) reported that AMF (*G*. *mosseae* and *G. etunicatum*) significantly increased contents of some metabolites (e.g., total phenols and proline) in walnut plants under DS. Moreover, AMF also promoted plant growth and nutrient acquisition in walnut plants (Thioye et al., 2022), and thereby improved the adaption of walnut plants in response to DS. The study of Liu et al. (2021) showed the an endophytic fungus (*Serendipita indica*) triggered the enhancement in superoxide dismutase (SOD), catalase (CAT), and peroxidase (POD) activities in walnut plants under soil water deficit, accompanied by the reduction of superoxide anion free radical (O_2_^•−^) and hydrogen peroxide (H_2_O_2_) levels, thus alleviating drought-induced oxidative burst. These results suggested that symbiotic fungi can alleviate oxidative burst in walnut under drought by activating antioxidant defense systems. However, it is unclear whether the dominant AMF strain, *D. spurca* (Huang et al., 2020), has similar functions in response to drought as *S*. *indica*.

Heat shock transcription factors (*Hsfs*) are key components of signal transduction and also regulate the response of genes to stress (Si et al., 2021). Moreover, *Hsfs* members such as *SPL7*, *HsfA1b*, *HsfA4a*, and *HsfA8* are involved in the homeostasis of reactive oxygen species (ROS) under DS conditions (Hoang et al., 2019). In addition, *Hsfs* can sense ROS in plant cells, and *Hsfs* are an important regulator to control oxidative burst under stress (Miller et al., 2008).

Although AMF has been shown to enhance drought tolerance in many plants, it is not clear whether and how a dominant strain, *D*. *spurca*, enhances drought tolerance in walnuts. We hypothesized that AMF could activate antioxidant defense systems and *Hsfs* transcription levels to alleviate oxidative damage caused by drought. Hence, the present study was performed to analyze effects of *D*. *spurca* on plant growth, antioxidant enzyme activities, antioxidant concentrations, transcription levels of *Hsfs*, ROS levels, and degree of membrane lipid peroxidation in leaves of walnuts subjected to DS.

## Materials and methods

### Plant culture, mycorrhizal inoculation, and soil water regimes

Walnut seeds of ‘Liaohe No. 1’ variety were pre-disinfected with 75% ethanol and germinated in autoclaved sands at room temperature. Subsequently, the seedlings having four leaves were transplanted into 2.4-L plastic pots containing 2.05 kg autoclaved mixture of sand and soil in the volume ratio of 1: 3. Mycorrhizal fungal inoculums were applied to the rhizosphere of walnut seedlings at the time of plant transplanting. The AMF-inoculated treatment (+AMF) received 150 g inoculum (23 spores/g) of *D*. *spurca* per pot, and the non-AMF-inoculated treatment (-AMF) received both 2 mL of 25 μm of inoculum filtrates and 150 g of autoclaved mycorrhizal inoculum per pot. The origin and propagation of the *D*. *spurca* strain were described in detail by Huang et al. (2020).

After plant transplanting, two soil moisture regimes (75% and 50% of the maximum soil water capacity) were performed according to the result of Li et al. (2020). The water content of potted soil was controlled at 75% of the maximum soil water capacity (well-watered, WW). After 7 weeks, half of the treated plants continued to maintain under WW conditions, and the other half of the treated plants was adjusted to 50% of the maximum soil water capacity (DS). The soil moisture was monitored by daily weighing, and the reduced water was supplemented immediately, so as to maintain the designed soil moisture condition. Such DS treatment was maintained for 6 weeks, and the seedlings were harvested. All the plants were grown in a greenhouse with a light density of 1360 lux, a relative air humidity of 66%, and a temperature of 28°C/22°C (day/night).

### Experimental design

The experiment was a completely randomized block design consisting of two factors: (i) *D*. *spurca* inoculation (+AMF) and non-inoculation (-AMF); and (ii) soil moisture regimes with WW and DS. A total of four treatments in the experiment were arranged, coupled with five replicates (two pots as a replicate) per treatment.

### Measurements of mycorrhizal development and plant growth

Stem diameter, plant height, and leaf number per plant were measured before harvesting. Shoot and root biomass was weighed after the harvest. Root mycorrhizas were stained using the trypan blue described by Phillips and Hayman (1970). Mycorrhizal fungal colonization degree (%) = (mycorrhizal colonized root length/total length of root segments examined) × 100. Hyphal length in the soil was determined as per the method of Bethlenfalvay and Ames (1987).

### Measurements of ROS levels and degree of membrane lipid peroxidation in leaves

Malondialdehyde (MDA, an indicator of the degree of membrane lipid peroxidation) concentrations in leaves were assayed by the thiobarbituric acid colorimetry (Sudhakar et al., 2001). H_2_O_2_ and O_2_^•─^ levels were assayed by the 1 mol/L KI colorimetric method and the hydroxylamine reaction, respectively (Li et al., 2022).

### Measurements of non-enzymatic antioxidant concentrations in leaves

Soluble protein concentrations in leaves were measured as per the protocol described by Bradford (1976). Ascorbic acid (ASC) and glutathione (GSH) in leaves were extracted by grinding 0.15 g of leaf samples with 6 mL of 5% trichloroacetic acid into a homogenate and centrifuging at 15,000×*g* for 10 min (Wu et al., 2006). ASC and GSH concentrations in the supernatant were measured according to the method described by Li (2009). In addition, the 1 mL supernatant was incubated with 0.5 mL of 60 mmol/L dithiothreitol for 10 min to reduce the dehydroascorbic acid (DHA). The reaction solution was then incubated with 5% trichloroacetic acid, 0.4% phosphoric acid, 0.5% bathophenanthroline, and 0.03% FeCl_3_, and the absorbance at 534 nm was measured for total ascorbic acid (TASC) concentrations. The DHA content was obtained by subtracting ASC from TASC.

### Measurements of antioxidant enzyme activities in leaves

Extraction and activity of CAT were carried out by UV spectrophotometry (Li et al., 2022). POD activity was determined using the guaiacol (0.05 mol/L) method (Li et al., 2022). Ascorbate peroxidase (APX) and glutathione reductase (GR) activities were assayed by the protocol outlined by Wu et al. (2006). Fe-SOD, Mn-SOD, and Cu/Zn-SOD activities were measured by the Enzyme-Linked Immunosorbent assay using the corresponding kit (ml902210, mll614100, and ml201168) (Shanghai Enzyme-link Biotechnology Co., Ltd., Shanghai, China), on the basis of the user manual.

### Measurements of expression levels of *JrHsfs* in leaves

Based on the identification of *Hsfs* in walnuts by Liu et al. (2020), the sequence of walnut *Hsfs* genes (*JrHsf03*, *JrHsf05*, *JrHsf20*, *JrHsf22*, and *JrHsf24*) was extracted from the walnut genome (https://www.ncbi.nlm.nih.gov/genome/?term=txid2249226 [orgn]). Primer sequences (**Table 1**) were designed using Primer premier 5.0. The TaKaRa MiniBEST plant RNA kits (9769; Takara, Dalian, China) were used to extract leaf total RNA according to the user manual. After checking the integrity and concentration, the RNA was reversely transcribed into cDNA using the PrimeScript™ RT reagent kits with gDNA Eraser (RR047A; Takara, Dalian, China). The cDNA was used as a template for qRT-PCR amplification using 18S-rRNA as a house-keeping gene. Prior to performing qRT-PCR, the selected primers and melting curves had been checked to determine the reliability of the relative quantification results. Real-time fluorescence quantitative expression analysis was performed using a fluorescent dye method with three biological replicates of each treatment, and relative expression of genes was calculated using the 2^-ΔΔCt^ method (Livak and Schmittgen, 2001).

**Table 1:** Specific primer sequences of genes used for qRT-PCR.

Gene name	Gene ID	Primer sequences (5’→3’)
*JrHsf03*	LOC109009449	F: TGCTTATGATGTCATGGCAGAGA
		R: TCCTCCTCTAAATCCACCCAAA
*JrHsf05*	LOC108997276	F: AGACTCCCCAATCAAGAGGAAAG
		R: CCGCAGCAAGGTTTTAGCA
*JrHsf20*	LOC108992254	F: AGGTTGTTCTTGAGCTTTCGATG
		R: GGTAGGTTTTGGTGAGGAATGG
*JrHsf22*	LOC109011524	F: GAACGGGGTTTGTAGTATGGTCTC
		R: GACACTTGGCTCGCACTTCTT
*JrHsf24*	LOC108989320	F: GAAGACGTACATGCTGGTGGAG
		R: TATGCTTGAAAAGTGTAGGGAGGAG
*18S-rRNA*	LIHL01052714.1_7	F: GGTCAATCTTCTCGTTCCCTT
		R: TCGCATTTCGCTACGTTCTT

### Data analysis

Statistical analysis was performed with two-factor analysis of variance, based on the SAS software 8.1v (SAS Institute Inc., Cary, NC, USA). The Duncan’s multiple range test at the 0.05% level was used to compare the significant difference among treatments.

## Results

### Root mycorrhizal colonization

No mycorrhiza was observed in the roots of walnut inoculated without *D*. *spurca*, and the degree of mycorrhizal colonization on the roots of walnut inoculated with *D*. *spurca* ranged from 62.9% to 73.5% (**Table 2**). Soil water deficit significantly inhibited the degree of root mycorrhizal colonization by 14.4%, compared to WW treatment. Soil drought treatment and AMF inoculation significantly interacted on root mycorrhizal colonization.

**Table 2:** Effects of AMF (*Diversispora spurca*) on root mycorrhizal colonization and plant growth performance of walnut under well-watered (WW) and drought stress (DS).

Treatments	Root mycorrhizal colonization (%)	Plant height(cm)	Stem diameter(mm)	Leaf number per plant	Biomass (g/plant)
WW+AMF	73.5 ± 3.8a	47.2 ± 4.8a	6.7 ± 0.4a	36.6 ± 3.2a	34.8 ± 1.6a
WW-AMF	0.0 ± 0.0c	36.5 ± 5.6b	6.0 ± 0.9ab	34.6 ± 3.0a	27.8 ± 2.0b
DS+AMF	62.9 ± 4.1b	36.2 ± 2.2b	5.3 ± 0.4b	33.4 ± 2.8a	27.7 ± 4.3b
DS-AMF	0.0 ± 0.0c	26.6 ± 7.8c	3.6 ± 0.8c	29.2 ± 2.6b	21.4 ± 1.7c
*Significance*					
DS	**	**	**	**	**
AMF	**	**	**	*	**
DS×AMF	**	NS	NS	NS	*

Data (means ± SD, n = 4) followed by different letters among treatments indicate significant differences at the 5% level. * P < 0.05; ** P < 0.01; NS, not significant at the 0.05 level.

### Plant growth responses

Drought treatment obviously inhibited the growth of walnut seedlings, while mycorrhizal introduction improved plant growth (**Table 2**). *D*. *spurca* inoculation only significantly increased plant height under WW by 29.3%, whereas it increased plant height, stem diameter, and leaf number per plant under DS significantly by 36.1%, 47.2%, and 14.4%, respectively. AMF-inoculated seedlings exhibited 25.2% significantly higher biomass under WW and 29.4% higher biomass under DS, compared with non-inoculated seedlings. A significant interaction between drought treatment and mycorrhizal inoculation occurred on biomass production.

### ROS levels

**Figure 1:**
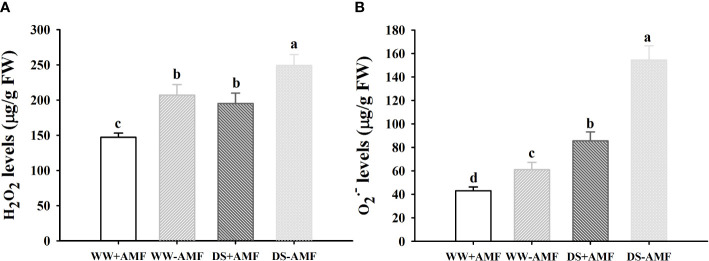
Effect of AMF (*Diversispora spurca*) on leaf H_2_O_2_ **(A)** and O_2_^•─^ **(B)** concentrations of walnut under well-watered (WW) and drought stress (DS). Data (means ± SD, *n* = 4) are significantly different (*P* < 0.05) if followed by different letters above the bars.

Drought treatment significantly induced an increase in leaf H_2_O_2_ and O_2_^•─^ concentrations by 20.3% and 152.6% in uninoculated plants and by 32.2% and 98.7% in inoculated plants (**Figures 1A, B**). However, the inoculated plants with *D*. *spurca* recorded significantly lower leaf H_2_O_2_ and O_2_^•─^ concentrations by 44. 5% and 41.7% under WW and by 27.7% and 80.2% under DS, respectively, compared with the uninoculated plants. A significant interaction between drought treatment and AMF inoculation occurred on O_2_^•─^ concentrations (**Table 3**).

**Table 3:** Significance of variables between AMF and non-AMF colonized walnut seedlings grown in well-watered (WW) and drought stress (DS).

Variables	DS	AMF	DS×AMF	Variables	DS	AMF	DS×AMF
H_2_O_2_	**	**	NS	Cu/Zn-SOD	**	**	NS
O_2_^•−^	**	**	**	CAT	**	**	**
MDA	**	**	f	**	POD	**	**	NS
Soluble protein	**	**	NS	APX	**	**	*
GSH	**	**	NS	GR	**	**	NS
ASC	**	**	NS	*JrHsf03*	**	**	NS
DHA	**	**	NS	*JrHsf05*	NS	**	**
TASC	**	**	NS	*JrHsf20*	**	**	NS
Mn-SOD	**	**	NS	*JrHsf22*	**	**	**
Fe-SOD	**	**	**	*JrHsf24*	**	**	NS

NS, not significant at the 0.05 level. *, *P* <0.05; **, *P* <0.01.

### Degree of membrane lipid peroxidation

**Figure 2:**
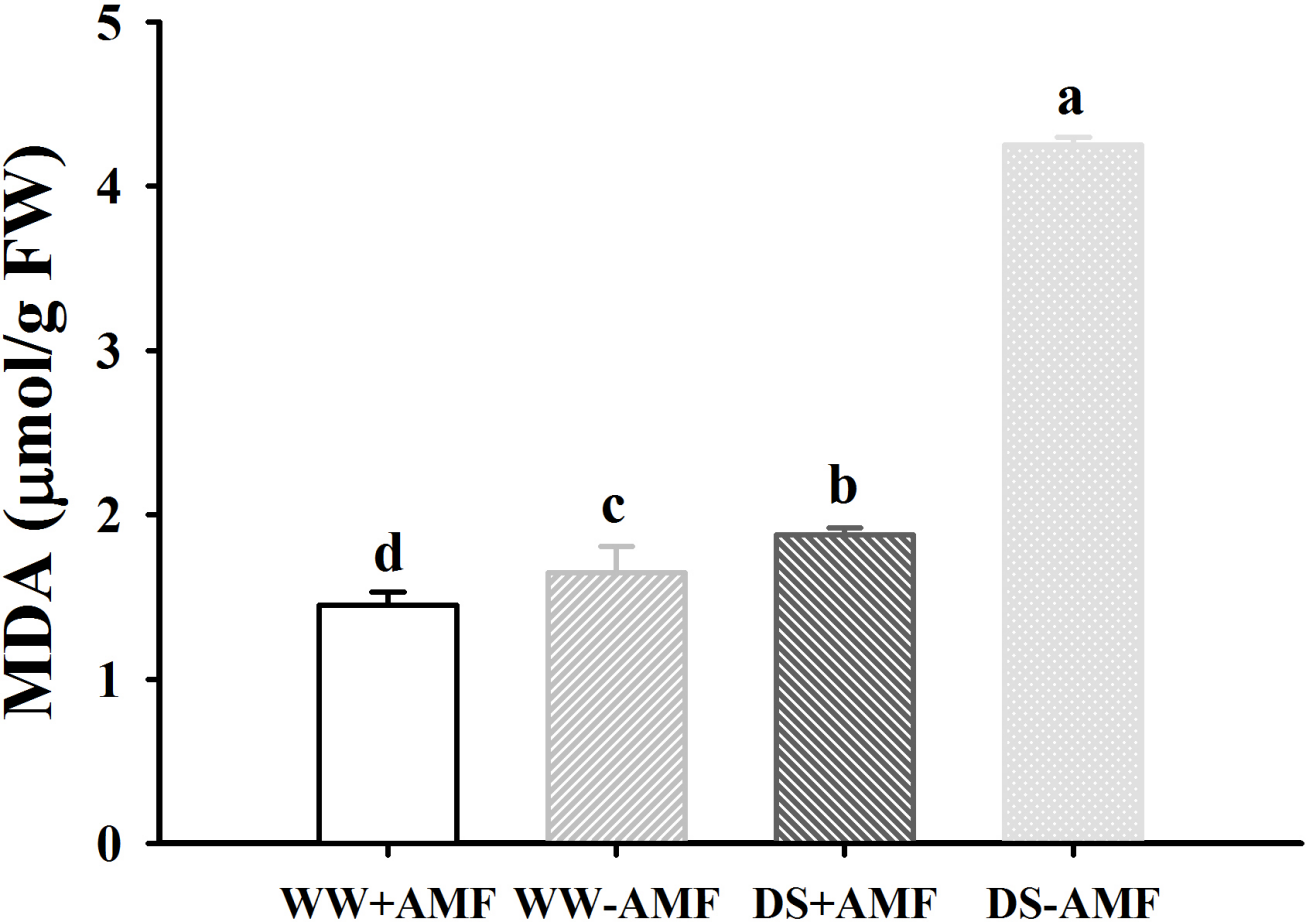
Effect of AMF (*Diversispora spurca*) on leaf malondialdehyde (MDA) concentrations of walnut under well-watered (WW) and drought stress (DS). Data (means ± SD, *n* = 4) are significantly different (*P* < 0.05), if followed by different letters above the bars.

The DS treatment significantly promoted MDA levels in both inoculated and uninoculated plants by 29.7% and 157.6%, respectively, relative to the WW (**Figure 2**). On the other hand, inoculated walnut plants showed significantly lower MDA levels by 13.8% under WW and 126.1% under DS. There was a significant interaction between drought treatment and AMF inoculation for MDA levels (**Table 3**).

### Non-enzymatic antioxidant concentrations

**Figure 3:**
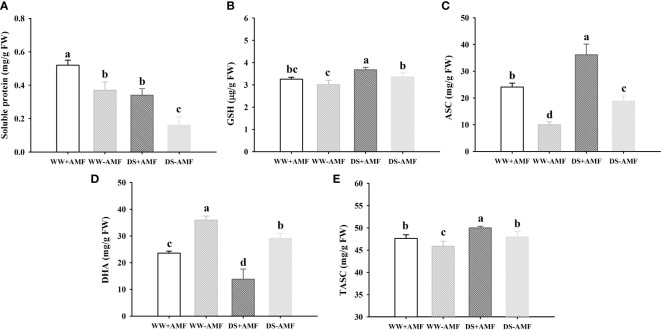
Effect of AMF (*Diversispora spurca*) on leaf soluble protein **(A)**, GSH **(B)**, ASC **(C)**, DHA **(D)**, and TASC **(E)** concentrations of walnut under well-watered (WW) and drought stress (DS). Data (means ± SD, *n* = 4) are significantly different (*P* < 0.05), if followed by different letters above the bars.

Compared to the WW treatment, the DS treatment triggered a distinct decrease in soluble protein and DHA concentrations in inoculated and uninoculated plants, but induced an increase in GSH, ASC and TASC concentrations in inoculated and uninoculated plants (**Figures 3A** **–E**). Under WW conditions, soluble protein, ASC and TASC concentrations were increased by 40.54%, 141.57% and 3.79% in inoculated plants, compared to uninoculated plants (**Figures 3A, C, E**). Under DS conditions, soluble protein, GSH, ASC and TASC concentrations of inoculated plants were increased by 112.50%, 9.52%, 91.89% and 4.17%, compared to that of uninoculated plants (**Figures 3A, B, C, E**). AMF inoculation caused the decrease in DHA concentrations by 52.46% and 110.78% under WW and DS, respectively, compared with non-AMF inoculation (**Figure 3D**). No significant interaction between drought treatment and AMF inoculation occurred on non-enzymatic antioxidants (**Table 3**).

### Antioxidant enzyme activities

**Figure 4:**
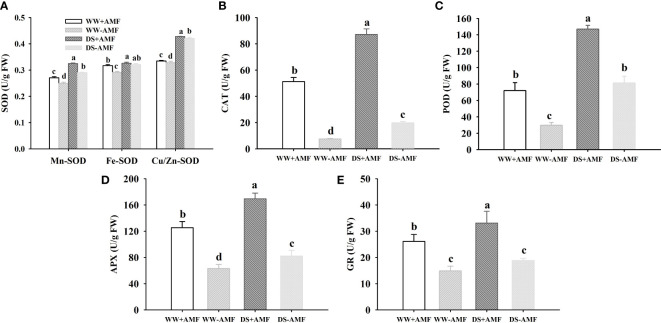
Effect of AMF (*Diversispora spurca*) on leaf superoxide dismutase (SOD) **(A)**, catalase (CAT) **(B)**, peroxidase (POD) **(C)**, ascorbate peroxidase (APX) **(D)**, and glutathione reductase (GR) **(E)** activities of walnut under well-watered (WW) and drought stress (DS). Data (means ± SD, *n* = 4) are significantly different (*P* < 0.05), if followed by different letters above the bars.

The DS treatment significantly increased various antioxidant enzyme activities compared to WW treatment, independent of AMF inoculation or not (**Figures 4A** **–E**). In addition, under WW, Mn-SOD, Fe-SOD, CAT, POD, APX and GR activities were increased by 8.8%, 8.9%, 570.2%, 142.3%, 98.7% and 76.0% in inoculated plants compared to uninoculated plants, respectively; under DS, Mn-SOD, Cu/Zn-SOD, CAT, POD, APX and GR activities were increased by 13.8%, 1.7%, 340.4%, 80.5%, 106.3% and 77.2% in inoculated plants compared to uninoculated plants, respectively. A significant interaction between DS and AMF treatment occurred on Fe-SOD, CAT, and APX activities (**Table 3**).

### *Hsfs* expression levels in leaves

**Figure 5:**
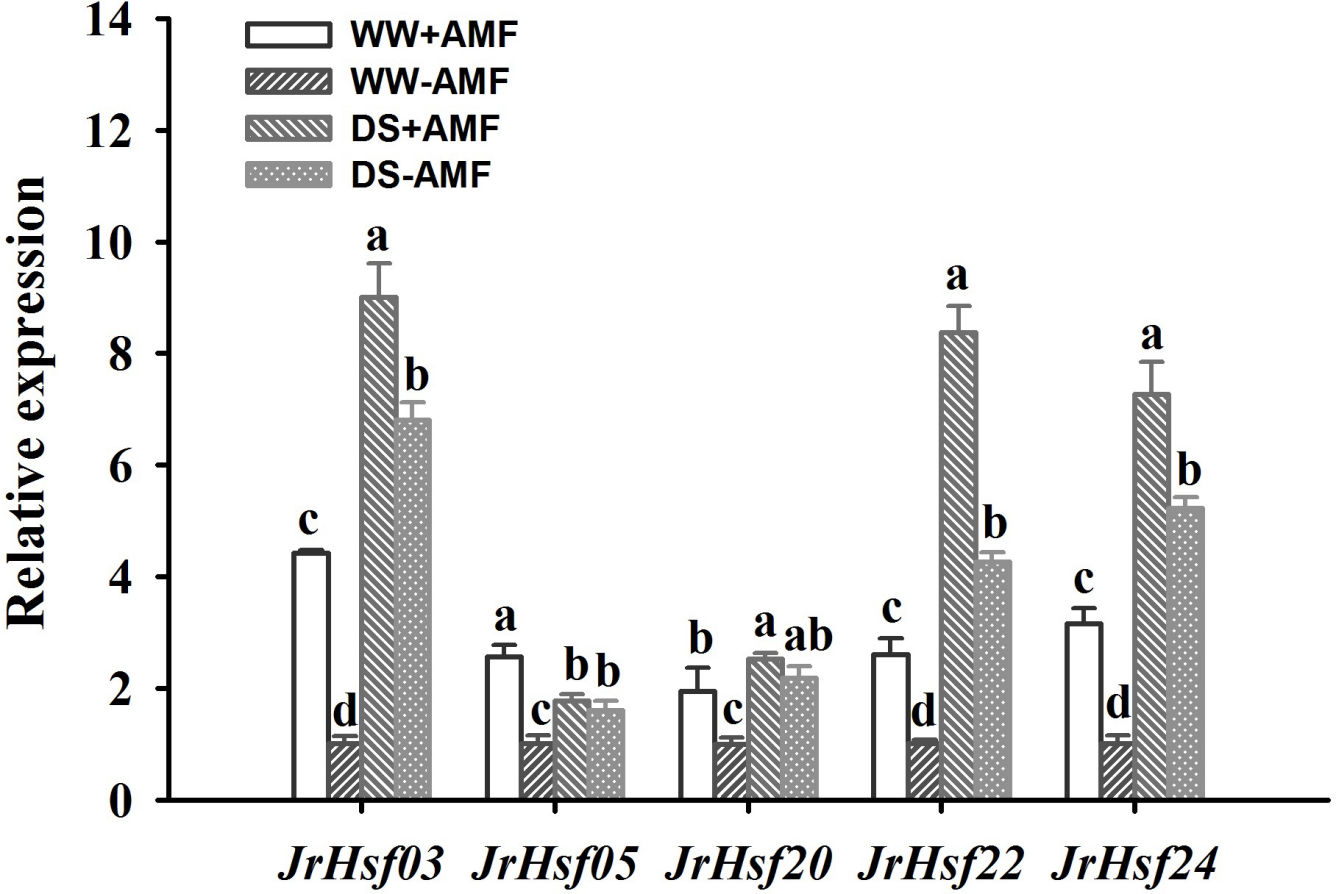
Effect of AMF (*Diversispora spurca*) on *Hsfs* gene expression levels in leaves of walnut under well-watered (WW) and drought stress (DS). Data (means ± SD, *n* = 3) are significantly different (*P* < 0.05), if followed by different letters above the bars.

Drought treatment up-regulated expressions of *JrHsf03*, *JrHsf20*, *Jrhsf22* and *JrHsf24* in inoculated and uninoculated walnut plants, compared to WW treatment (**Figure 5**). However, DS also induced *JrHsf05* expressions in uninoculated plants, but down-regulated *JrHsf05* expressions in inoculated plants. AMF inoculation significantly up-regulated expressions of *JrHsf03*, *JrHsf05*, *JrHsf20*, *JrHsf22*, and *JrHsf24* under WW by 4.42-fold, 2.57-fold, 3.15-fold, 2.60-fold, and 1.95-fold, respectively, compared to non-AMF treatment; under DS, AMF up-regulated expressions of *JrHsf03*, *Jrhsf22*, and *JrHsf24* by 1.32-fold, 1.96-fold, and 1.39-fold, respectively, compared to non-AMF inoculation, with no effect on the expression of *JrHsf05* and *JrHsf20*. A significant interaction between drought treatment and AMF inoculation occurred on *JrHsf05* and *JrHsf22* expression (**Table 3**).

## Discussion

### Soil drought inhibited mycorrhizal colonization, while AMF still promoted walnut growth under drought

Our study indicated that the DS treatment reduced the degree of root colonization by *D*. *spurca* in walnut seedlings. Similar result was reported in trifoliate orange (Liang et al., 2021) and peanut (Bi et al., 2021). Such reduction of mycorrhizal colonization under DS is due to the decrease in roots, host’s carbohydrates, and root exudates by DS, thus inhibiting spore germination and mycorrhizal colonization (Tyagi et al., 2017). Inoculation with *D*. *spurca* significantly promoted plant growth performance of walnut seedlings, which is attributed to the fact that AMF enhanced the uptake of mineral nutrients such as P, Zn and Cu, along with water absorption by mycorrhizal extraradical hyphae (Cheng et al., 2021).

### AMF activated enzymatic and non-enzymatic antioxidant defense systems to mitigate oxidative burst under drought

Under stress, plants produce electron overflow in chloroplasts, mitochondria, peroxisomes, and plasma membranes, and thus lead to excess accumulation of ROS such as H_2_O_2_ and O_2_^•─^, which thus triggers oxidative damage (Cao et al., 2022). This study showed that the soil drought triggered oxidative burst (H_2_O_2_ and O_2_^•─^) in leaves of mycorrhizal and non-mycorrhizal walnut plants, thus increasing the degree of membrane lipid peroxidation, in accordance with increased MDA levels. However, *D*. *spurca*-inoculated walnut plants presented significantly lower ROS and MDA levels than uninoculated plants, suggesting that inoculated plants suffered relatively lower oxidative damage than uninoculated plants (Abd_Allah et al., 2015). This finding is consistent with that on trifoliate orange (Zhang et al., 2020) and lettuce (Kohler et al., 2009).

Soil drought causes oxidative damage to plants, while plants also have enzymatic (e.g., SOD, POD, and CAT) and non-enzymatic (e.g., soluble protein, ASC, and GSH) antioxidant defense systems to reduce ROS levels (Verma et al., 2019). Soluble proteins are involved in the metabolic process and are related to the water holding capacity of cells and the protective role in cell membranes (Lu et al., 2012). Our study showed that soil drought treatment inhibited soluble protein concentrations in walnut leaves, while AMF inoculation promoted soluble protein concentrations, suggesting that the inoculated plants had stronger water retention of cells and protective effect on cell membranes than uninoculated plants. Cao et al. (2022) found that *Piriformospora indica*, but not *G*. *versiforme*, also significantly increased leaf and root soluble protein concentrations in Satsuma mandarin, under cold temperature, but not favorable temperature conditions, implying that AMF-mediated changes in soluble protein depend on AMF species, host genotypes, and environmental stresses.

ASC-GSH cycle regulates the balance of redox state of plant cells and is an important pathway for ROS removal (Miller et al., 2010). Meanwhile, GSH maintains cell function and regulates the state of sulfhydryl groups, and ASC as an electron donor participates in substance transformation (Noctor, 2006). In this cycle, ASC is first oxidized to monohydroascorbic acid (MDHA), in which APX utilizes ASC as an electron donor to remove H_2_O_2_ (Wang et al., 2012). In our study, inoculated walnut plants under two soil moisture conditions showed significantly higher ASC and TASC concentrations and stronger APX activities than uninoculated plants, implying that AMF activates ASC to scavenge more H_2_O_2_ of the host caused by drought. In addition, AMF inoculation also increased GSH concentrations of walnut plants while decreased DHA concentrations under DS. It is known that MDHA can undergo disproportionation reaction to produce ASC and DHA (Qadir et al., 2022). DHA uses GSH as the substrate to generate GSSG and ASC under the action of dehydroascorbate reductase, and GSSG further combines with NAD(P)H as an electron donor to generate GSH under the action of GR (Li et al., 2010). Lower DHA levels and higher GSH levels and GR activity in mycorrhizal plants under DS mean that mycorrhizal plants convert more DHA to ASC and modulate more accumulation of GSH under the action of GR, as compared with non-mycorrhizal plants. Al-Arjani et al. (2020) also reported the elevation in GR and APX activities in *Ephedra foliata* plants after inoculated with AMF under DS. Saroy and Garg (2021) also observed that *Rhizoglomus intraradices* distinctly increased APX and GR activities and GSH, TASC, ASC, and GSSH concentrations in two pigeon pea genotypes under Ni stress. *Glomus viscosum*-inoculated *Cynara scolymus* plants also exhibited higher ASC and GSH concentrations than non-inoculated plants, along with elevated APX activities, for resisting the fungal pathogen *Verticillium dahliae* (Villani et al., 2021). These results indicate that mycorrhizal plants have a stronger ASC-GSH cycle to remove more H_2_O_2_ induced by stresses than non-mycorrhizal plants, thus maintaining lower oxidative damage.

In ROS scavenging enzymes, SOD catalyzes O_2_^•─^ to H_2_O_2_; generated H_2_O_2_ is then removed by POD and CAT (Qadir et al., 2022). In our study, three SODs, CAT, and POD activities were enhanced by DS, indicating that the enzymatic antioxidant defense system in walnut plants was activated in response to drought. Additionally, mycorrhizal walnut plants recorded higher POD, CAT, Mn-SOD, and Zn-SOD activities than non-mycorrhizal plants under two soil moisture regime conditions. Similar results were reported in *Citrus sinensis* inoculated with three different AMF species (Li et al., 2022). He et al. (2020) further found the induced expression of *PtMn-SOD*, *PtCAT1*, and *PtPOD* genes in trifoliate orange by *F*. *mosseae* under DS. These results suggest that AMF enhanced enzymatic antioxidant defense system to mitigate oxidative burst in response to DS.

### AMF activated expressions of some Hsfs members such as *JrHsf03*, *JrHsf22*, and *JrHsf24* under drought

Our study revealed that soil drought induced transcriptional levels of *JrHsf03*, *JrHsf20*, *Jrhsf22*, and *JrHsf24* in walnut plants, independent on mycorrhizal presence. It suggests that *Hsfs* of walnut can respond to DS, not limited to heat stress, which is consistent with the results of Liu et al. (2020) in *Hsfs* of walnut under DS, heat stress, and salt stress. Similar responses of *Hsfs* to DS were also observed in mulberry (Zhai and Zhu, 2021) and arabidopsis (Tan et al., 2015). Moreover, We also firstly observed that *JrHsf03*, *JrHsf05*, *JrHsf20*, *JrHsf22*, and *JrHsf24* were up-regulated by AMF inoculation under WW, while only *JrHsf03*, *JrHsf22*, and *JrHsf24* were induced by AMF inoculation under DS, indicating that AMF-mediated response of *JrHsfs* depends on *Hsfs* types. It is not clear whether *JrHsf03*, *JrHsf22*, and *JrHsf24* are specifically induced by AMF, which needs to be confirmed by additional studies. However, Gaude et al. (2012) reported the inhibited expression of *HsfB3* in arbuscule-containing cortical cells of mycorrhizal roots versus cortical cells of non-mycorrhizal roots with 3.2-fold after three weeks of inoculation in roots of *Medicago truncatula* plants. These heat shock factors were suppressed during the initial mycorrhizal colonization (Gaude et al., 2012). Nevertheless, our study was performed for 13 weeks along with soil drought, mycorrhizal colonization had already been established, and thus this suppression may be relieved. In addition, *Hsfs* members are redox-sensitive transcription factors sensing ROS, transducing and amplifying the ROS signal by various proteins and transcription factors (e.g., WRKY) (Miller et al., 2008). In walnut plants, *Hsfs* may be associated with the signaling pathways of abscisic acid and Ca^2+^ that regulate ROS production (Mohanta et al., 2018; Liu et al., 2020). Hence, it is concluded that AMF activated some *Hsfs* members such as *JrHsf03*, *JrHsf22*, and *JrHsf24* to regulate ROS production, but additional evidence needs to be presented. In addition, most of *Hsfs* members are expressed highly in roots than other tissues (Dossa et al., 2016), and mycorrhizal colonization firstly occurs in roots. More work needs to focus on the responsive pattern of root *Hsfs* to AMF colonization under drought, how AMF-initiated *Hsfs* trigger the antioxidant defense system, and whether AMF’s *Hsfs* are also involved in this response.

## Conclusion

In short, our study confirmed that an arbuscular mycorrhizal fungus, *D. spurca*, could promote growth performance of walnut plants exposed to DS. In the meantime, *D*. *spurca* activated antioxidant defense systems (e.g., enzymatic defense system and ASC-GSH cycle) and transcription levels of three *Hsfs* to alleviate oxidative burst. This study firstly provides insights into the role of AMF-regulated responses of *Hsfs* in possibly mitigating ROS burst. However, future work needs to focus on how mycorrhizal fungi initiate host or fungal *Hsfs* to mitigate oxidative burst under drought.

## Data availability statement

The original contributions presented in the study are included in the article/supplementary material. Further inquiries can be directed to the corresponding authors.

## Author contributions

W-YM, Y-JX, and Q-SW designed the experiment. W-YM, Q-YQ, and Y-NZ prepared the materials for the experiment. W-YM, Q-YQ, Y-JX, and Y-NZ conducted the experiment. W-YM and Q-YQ analyzed the data. W-YM wrote the manuscript. KK, BG, AH, A-BA-A, KA, EA, and Q-SW revised the manuscript. All authors contributed to the article and approved the submitted version.

## Funding

This work was supported by the Open Fund in Hubei Key Laboratory of Economic Forest Germplasm Improvement and Resources Comprehensive Utilization, Hubei Collaborative Innovation Center for the Characteristic Resources Exploitation of Dabie Mountains, Huanggang Normal University (202019604), the Hubei Province ‘14th Five-Year’ Major Science and Technology Aid Tibet project (SCXX-XZCG-22016), and 2021 Undergraduate Innovation and Entrepreneurship Training Program of Yangtze University (Yz2021328). The authors are grateful to their sincere appreciation to the Researchers Supporting Project Number (RSP-2021/134), King Saud University, Riyadh, Saudi Arabia.

## Acknowledgement

This work was supported by the Open Fund in Hubei Key Laboratory of Economic Forest Germplasm Improvement and Resources Comprehensive Utilization, Hubei Collaborative Innovation Center for the Characteristic Resources Exploitation of Dabie Mountains, Huanggang Normal University (202019604), the Hubei Province ‘14th Five-Year’ Major Science and Technology Aid Tibet project (SCXX-XZCG-22016), and 2021 Undergraduate Innovation and Entrepreneurship Training Program of Yangtze University (Yz2021328). The authors are grateful to their sincere appreciation to the Researchers Supporting Project Number (RSP-2021/134), King Saud University, Riyadh, Saudi Arabia.

## Conflict of interest

The authors declare that the research was conducted in the absence of any commercial or financial relationships that could be construed as a potential conflict of interest.

## Publisher’s note

All claims expressed in this article are solely those of the authors and do not necessarily represent those of their affiliated organizations, or those of the publisher, the editors and the reviewers. Any product that may be evaluated in this article, or claim that may be made by its manufacturer, is not guaranteed or endorsed by the publisher.
